# Production and Bioconversion Efficiency of Enzyme Membrane Bioreactors in the Synthesis of Valuable Products

**DOI:** 10.3390/membranes13070673

**Published:** 2023-07-16

**Authors:** Bandana Padhan, Madhubanti Ray, Madhumita Patel, Rajkumar Patel

**Affiliations:** 1 Department of Biotechnology, School of Life Science and Biotechnology, Adamas University, Kolkata 700126, West Bengal, India; miki.bandana@gmail.com (B.P.);; 2 Department of Chemistry and Nanoscience, Ewha Womans University, 52 Ewhayeodae-gil, Seodaemun-gu, Seoul 03760, Republic of Korea; 3 Energy & Environmental Science and Engineering (EESE), Integrated Science and Engineering Division (ISED), Underwood International College, Yonsei University, 85 Songdogwahak-ro, Yeonsugu, Incheon 21938, Republic of Korea

**Keywords:** enzyme membrane bioreactor, oligosaccharides, oligodextran, inulin, lignin

## Abstract

The demand for bioactive molecules with nutritional benefits and pharmaceutically important properties is increasing, leading researchers to develop modified production strategies with low-cost purification processes. Recent developments in bioreactor technology can aid in the production of valuable products. Enzyme membrane bioreactors (EMRs) are emerging as sustainable synthesis processes in various agro-food industries, biofuel applications, and waste management processes. EMRs are modified reactors used for chemical reactions and product separation, particularly large-molecule hydrolysis and the conversion of macromolecules. EMRs generally produce low-molecular-weight carbohydrates, such as oligosaccharides, fructooligosaccharides, and gentiooligosaccharides. In this review, we provide a comprehensive overview of the use of EMRs for the production of valuable products, such as oligosaccharides and oligodextrans, and we discuss their application in the bioconversion of inulin, lignin, and sugars. Furthermore, we critically summarize the application and limitations of EMRs. This review provides important insights that can aid in the production of valuable products by food and pharmaceutical industries, and it is intended to assist scientists in developing improved quality and environmentally friendly prebiotics using EMRs.

## 1. Introduction

**Scheme 1:**
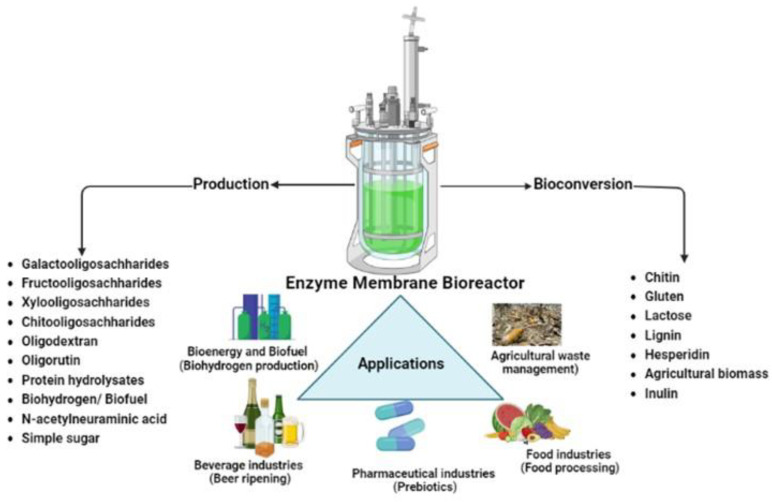
Production and bioconversion of various valuable products using enzyme membrane bioreactors (EMRs).

The downstream processing during purification is a time-consuming process in product synthesis using conventional bioreactors. Furthermore, the accurate separation of products from mixtures of by-products often involves the use of hazardous chemicals (organic solvents, chelating agents such as ethylenediaminetetraacetic acid (EDTA), or iminodiacetic acid), which may pose risks to human health and the environment. These chemicals are typically used in traditional separation techniques, such as solvent extraction or chromatography, to achieve high purity and specificity in product isolation [1,2]. However, in recent years, alternative sustainable processes, such as the use of enzyme membrane bioreactors (EMRs), Scheme 1 have attracted attention. EMRs combine the advantages of membrane separation and enzymatic conversion in a single system [3], and they enable accurate product purification via a membrane that selectively separates intermediate molecular weight products to address undesirable manufacturing concerns [4,5,6].

EMRs have found application in several fields, combining an efficient biocatalyst with a membrane separator to purify products in upstream and downstream processes [3,7,8]. Various economic types of EMRs, particularly size-exclusion EMRs, have attracted considerable attention, particularly in the agro-food, energy, and pharmaceutical industries [9,10]. EMRs offer several advantages in terms of enzyme recycling and stability. They provide a protective environment for enzymes, shielding them from harsh conditions such as high temperatures or extreme pH values, ensuring prolonged enzyme activity that, in turn, results in the improved performance and efficiency of biocatalytic processes [11,12,13]. Additionally, enzymes can be retained within the reactor using selective membranes, thereby preventing their loss and enabling their reuse, which reduces enzyme costs and increases the efficiency of the overall process. The membranes used in bioreactors enhance mass transfer between the reaction mixture and enzymes [14]. The controlled porosity of the membrane enables an efficient diffusion of substrates and products, optimizes reaction kinetics, and improves the overall performance of the biocatalytic process. Enzymes immobilized on the membrane surface create a large catalytic interface, resulting in increased reaction rates and higher productivity compared with traditional batch processes. Moreover, the modular nature of membrane systems enables the integration of multiple enzymatic reactions, making them versatile platforms for biotransformation [15]. A major limitation of EMRs is membrane fouling caused by the accumulation of impurities or enzyme deactivation, which can lead to reduced performance and increased maintenance requirements. Additionally, in some cases, the diffusion of large substrates or high substrate concentrations through the membrane can be challenging, thus limiting the reaction efficiency [5,10,16]. Moreover, depending on the operating conditions and enzyme properties, the membrane can degrade over time, affecting its performance and lifespan. Compared with traditional batch processes, the initial investment and maintenance costs of EMRs can be higher because of the need for specialized membranes and equipment. For example, lipolytic membranes were developed by coupling two industrial lipase enzymes onto a polyvinylidene fluoride (PVDF) flat sheet membrane, which have self-cleaning with high enzyme activity, effective fouling removal, and good reusability and separation applications [17].

Sitanggang et al. [3] conducted a comprehensive review of the design, application, and limitations of EMRs, focusing specifically on an EMR model for oligosaccharides and fructooligosaccharides production. However, further exploration is necessary to discuss the application of EMRs in the production of various products and the design of membranes with enzymes. In this review, we present the latest advancements in this field, as researchers continue to explore various directions every year. We summarize the development of EMRs and highlight their unique properties, which enable the production of diverse products. Additionally, we discuss the expanding applications of EMRs in different industries and highlight recent articles on various EMR designs. By examining the advantages, disadvantages, and processing methods of each EMR model, this review can serve as a reference for scientists and aid in the development of novel prebiotics and drugs.

## 2. Enzyme Membrane Synthesis and Challenges

### 2.1. Enzyme Membrane Synthesis

Enzyme membrane synthesis involves the immobilization or encapsulation of enzymes onto the surface or within the pores of a membrane. The process typically consists of multiple steps, each serving a specific purpose. The first step is to select an appropriate base membrane material that possesses the desired properties such as high porosity, mechanical strength, chemical stability, and compatibility with the enzymatic reaction [18,19]. Common base membrane materials include polymeric membranes (e.g., PVDF, cellulose acetate, and polyethersulfone) and inorganic membranes (e.g., ceramic or glass membranes) [20,21,22]. Polymeric membranes, such as polyamide, polyethersulfone, polypropylene, and PVDF, are widely used due to their flexibility, cost-effectiveness, and compatibility with a wide range of applications. Ceramic membranes, such as alumina, zirconia, and titania, exhibit excellent chemical and thermal stability, making them suitable for harsh operating conditions. Metal membranes, including stainless steel and titanium, offer high mechanical strength and resistance to fouling and chemical degradation. They are commonly used in applications that require high-temperature and high-pressure operations.

The surface of the base membrane is often modified to introduce functional groups that promote enzyme immobilization [23,24]. This step can involve chemical treatments, such as plasma activation and surface functionalization with specific linkers, or physical treatments such as ultraviolet or gamma irradiation. During the attachment step, the enzyme is covalently bonded to the modified membrane surface using various techniques, including crosslinking, grafting, and direct chemical bonding. In particular, crosslinking is a technique used to immobilize enzymes on membranes by creating covalent bonds between enzyme molecules and the membrane surface [25,26,27]. In a study, lipolytic membranes were developed by coupling two industrial lipase enzymes onto a polyvinylidene fluoride (PVDF) flat sheet membrane. The effects of three numerical factors (enzyme concentration, impregnation time, and irradiation dose) on enzyme activity were investigated using response surface methodology (RSM). The optimized conditions resulted in a maximum enzyme activity [17]. This process involves the use of bifunctional molecules that can form bridges between the enzyme and the membrane. Various bifunctional molecules, including bisdiazotized benzidine, glyoxal, hexamethylenediamine, and glutaraldehyde, can be used for enzyme immobilization on membranes as they contain reactive groups that can react with specific functional groups on both enzyme and membrane surfaces, resulting in the formation of covalent bonds [28,29]. 

As an alternative to surface attachment, enzymes can be encapsulated or entrapped in membrane pores, which is a process that involves the incorporation of enzymes into the membrane matrix during membrane synthesis. There are several methods for enzyme entrapment in the membrane, including physical entrapment, covalent attachment, and crosslinking. In the physical entrapment method, enzymes are mixed with the membrane material during its preparation [30]. The enzymes become physically trapped within the membrane structure, either in the pore spaces or within the polymer matrix. Enzymes can be covalently attached to the membrane surface or the functional groups present in the membrane material. This method provides stronger enzyme entrapment but requires appropriate chemistry to create covalent bonds. Enzymes can be crosslinked within the membrane by using suitable crosslinking agents or by modifying the enzyme or membrane with crosslinkable functional groups. Crosslinking enhances enzyme stability and prevents enzyme leakage [31]. To enhance the stability and longevity of the immobilized enzymes, crosslinking agents, such as glutaraldehyde, can be used to chemically stabilize enzyme structures and prevent leaching from the membrane. 

The next essential step is blocking to prevent nonspecific adsorption or fouling, during which the remaining surface area of the membrane is not occupied by enzymes. This step involves the introduction of blocking agents or polymers that reduce the adsorption of unwanted species and enhance the selectivity of enzyme reactions. Different types of blocking agents or polymers can be used depending on the specific application and requirements of the enzymatic reaction. Some common examples include bovine serum albumin, casein, polyethylene glycol, and various surfactants. These blocking agents are typically added to the reaction system in a concentration sufficient to occupy the unoccupied surface area and reduce unwanted adsorption [32]. Following enzyme immobilization, the membrane may be subjected to post-treatment or conditioning to remove unbound or weakly bound enzymes, which is a process that involves washing, rinsing, or soaking the membrane in suitable buffers or solutions [33,34]. 

### 2.2. Challenges for Enzyme Membrane Bioreactor 

Fouling is a major challenge encountered in membrane processes, where unwanted materials accumulate on the membrane surface and impede its performance. Particles in the feed solution can deposit on the membrane surface, reducing permeability and causing flux decline. Scaling occurs when inorganic salts precipitate and form a layer on the membrane, leading to reduced performance. Biological fouling involves the growth of microorganisms, such as bacteria and algae, on the membrane surface, resulting in biofilm formation and reduced permeability. Organic fouling is caused by the deposition of organic compounds on the membrane surface, reducing permeability and requiring more frequent cleaning [35]. 

Another challenge of EMB is membrane selection with appropriate pore size or molecular weight cutoff (MWCO). To recycle enzymes in an EMR, it is essential to choose a membrane with an appropriate pore size or MWCO that allows the reactants to freely diffuse through while retaining the enzymes within the reactor. By maintaining the enzymes within the reactor, they can be reused for multiple reaction cycles, improving efficiency and reducing the need for enzyme replenishment [36]. The design of the EMBR system considers the selection of an appropriate membrane with a pore size or MWCO that strikes a balance between allowing reactant diffusion and enzyme retention. Careful consideration and characterization of the enzyme and membrane properties are essential to ensure compatibility and optimal performance in EMRs [36,37]. Multichannel tubular ceramic membranes with different MWCO geometries can efficiently separate fructooligosaccharides (FOSs) [37]. In another study, a polyether sulfone ultrafiltration (UF) membrane with an MWCO of 20 kDa was designed to retain or separate oligodextrins [36].

Operating parameters such as feed flow rate, transmembrane pressure, temperature, pH and chemical compatibility, membrane configuration, pore size, etc. are the conditions under which membrane separation processes are conducted. These parameters can significantly influence the performance and efficiency of the membrane system. Transmembrane pressure (TMP) is an important operating parameter that affects the flux and selectivity of the membrane process. Higher TMP can enhance the permeate flux but may also increase the risk of membrane fouling. Crossflow velocity refers to the velocity of the feed solution tangentially to the membrane surface [38]. Adequate crossflow velocity helps reduce fouling by preventing the accumulation of foulants on the membrane surface. pH and temperature affect the stability and performance of both the membrane and the feed solution. Optimal pH and temperature conditions are often required for specific separations [39]. The concentration of solutes in the feed solution affects the driving force for mass transfer across the membrane. Higher solute concentrations may increase the osmotic pressure and affect the performance of the membrane process. The proper arrangement of the membrane, enzyme immobilization techniques, and reactor configuration (such as batch, continuous, or hybrid systems) affect mass transfer, substrate conversion, and enzyme stability [40,41]. The maximum conversion rate in the reverse hydrolysis of glucose to gentiooligosaccharides was achieved at pH 7.0 and 40 °C [39], and the conversion of chitin was reported to be effective using this biocatalytic UF membrane reactor [42].

Membrane filtration operations play a crucial role in EMR performance. The choice of filtration operation parameters, such as dead-end vs. crossflow filtration, membrane types and configurations, transmembrane pressure, crossflow velocity, and operating temperature, can significantly impact EMR efficiency and overall performance. During dead-end filtration, the feed solution passes directly through the membrane, resulting in the accumulation of solutes and particles on the membrane surface [43,44], which can cause fouling and decrease the filtration efficiency over time. Therefore, dead-end filtration is generally suitable for low fouling solutions, or when frequent membrane cleaning is possible. Crossflow filtration involves tangential flow of the feed solution across the membrane surface, which generates shear stress that helps prevent fouling by continuously sweeping away particles and solutes from the membrane surface. Crossflow filtration is typically preferred for EMR applications because it offers higher filtration efficiency and longer operation times between cleaning cycles [37,45].

## 3. Production of Different Valuable Product Using EMR

### 3.1. Production of Oligosaccharides

EMR application may lower the high operational expenses related to enzyme inactivation and removal. Prebiotic carbohydrates are galactooligosaccharides (GOS) that are used in the food sector and are enzymatically produced from lactose for technological or nutritional benefits [5]. The integration of selective mass transport and enzymatic synthesis has been achieved using a membrane bioreactor, which facilitates the targeted removal of reaction products and enhances the conversion of product inhibited or thermodynamically unfavorable reactions. Although crossflow velocity helps mitigate fouling by continuously sweeping away foulants, it cannot completely eliminate. Proper control of the crossflow velocity and pressure helps minimize fouling and extend the operational lifetime of EMRs. It is important to strike a balance between sufficiently high crossflow velocities to prevent fouling and maintain flux rates while avoiding excessive pressure that may compromise enzyme activity and membrane integrity [5]. The study focused on the synthesis of GOS from lactose solutions with a concentration of 150 g·L^−1^ at 40 °C, using a β-galactosidase enzyme at a concentration of 10 U·mL^−1^ and cellulose acetate membranes for sugar fractionation. The performance of the bioreactor was assessed by varying the pressure (20 and 24 bar) and crossflow velocity (1.7, 2.0, and 2.4 m·s^−1^). Integrating simultaneous GOS synthesis and production fractionation increased GOS production by 60% compared to reactions conducted without permeation. The presence of the enzyme and high total sugar concentration led to intricate selective mass transfer characteristics. In the absence of the enzyme, the membrane demonstrated tight UF properties, enabling the permeation of mono- and disaccharides while retaining only 25% of trisaccharides. During the simultaneous synthesis and fractionation process, the membrane retained GOS-3 completely while retaining GOS-2 and monosaccharides at rates of 80% and 40%, respectively. Depending on the complexity of the membrane bioreactor system, it has been observed that increasing the crossflow velocity by 18% can have a significant impact on reducing the total resistance to permeation, which can be a promising approach to mitigate membrane fouling and enhance the performance of membrane bioreactors [5].

GOS have been synthesized in EMRs using soluble biolactate N5 (a commercially available enzymatic product from *Bacillus circulans*) [46]. The steady-state performance of the EMR was investigated by varying the residence time (1.1–2.8 h) and enzyme load (17–190 U·g^−1^) while maintaining fixed operational conditions, such as temperature (50 °C), pH (6.0), lactose feed concentration (300 g·kg^−1^), and recirculation flow-rate (0.18 m^3^·h^−1^). Although a stirred tank reactor achieved a higher GOS yield with higher degree of polymerization (DP3-6) (approximately 38% of the total carbohydrate basis) than the EMR (24–33%), EMRs demonstrate stable catalytic performance over an extended operating period (>120 h) without significant deterioration in product quality [46].

**Figure 1:**
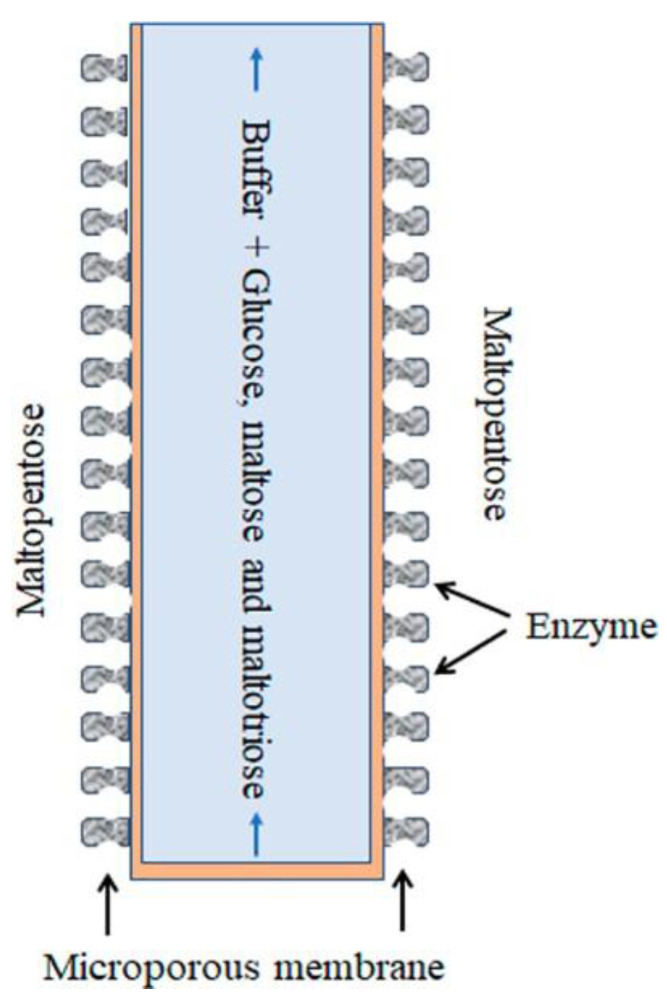
Membrane of a typical enzyme membrane bioreactor with immobilized enzymes [47].

Post-treatment is necessary for batch enzymatic hydrolysis during the production of glucose syrup without enzymes or non-hydrolyzed components. Membrane-based procedures represent alternative methods that do not require chemicals (Figure 1) [47]. In situ product recovery (ISPR) combines membrane and enzymatic hydrolysis. ISPR process investigations focus on the efficiency of the continuous feeding of substrates and enzymes with higher stability and resilience to abrupt changes in operational factors during enzymatic hydrolysis. The effectiveness of producing glucose syrups by enzymatic hydrolysis and membrane-based processes is influenced by temperature and pH. Most glucose syrups are produced using response surface methodology without the continuous feeding of substrates or enzymes. Reactor reuse reduces the cost of purchasing fresh membranes and enzymes.

Linear fructans known as FOSs comprise (2,5)-fructose units connected to a terminal glucose residue [48]. Owing to their sweetness, low-calorie value, and prebiotic characteristics, FOSs are extensively exploited as food and feed additives. Recombinantly synthesized fructosyltransferase produced by *Kluyveromyces lactis* produced FOSs in an enzyme solution devoid of cells. When glucose accumulates as a by-product of enzyme catalysis and eventually limits FOS formation, EMRs can be used to continuously remove glucose while simultaneously replenishing sucrose. Furthermore, FOSs have been reported to be synthesized using transfructosylating sucrose (FTases), which have hydrolytic and transfructosylating activities [49]. To achieve maximum FOS conversion, FTases must be deactivated or eliminated from a reactor. An integrated ultrafiltration–diafiltration–concentration technique has been used to purify FOSs and reuse FTases, wherein FOS purity is improved by removing monosaccharides from the reaction mixture. FOSs were extracted from the FTase-free FOS solution using a constant-volume diafiltration technique with an NF5 nanofiltration membrane, and a DL nanofiltration membrane was used to extract FOSs and sucrose from the diafiltration permeate. Higher FOS production has been achieved using an EMR via a semi-continuous production process combined with probiotic bacterium *Bacillus coagulans* fermentation than via the batch process [37]. Using multi-channel tubular ceramic membranes with different geometries and MWCOs enables flexibility in optimizing filtration performance for diverse applications. This flexibility allows for the efficient and reliable separation of FOSs, meeting the specific requirements of various industries. The fouling issue caused by protein accumulation can be effectively controlled by regulating filtration parameters such as flow rate, transmembrane pressure, and membrane characteristics [37]. Similarly, enzyme-free FOSs can be produced when FTase is immobilized on resin carriers [43]. Fed-batch fermentation with the probiotic bacterium Bacillus coagulans and FTase has been used to simultaneously convert residual sucrose. This approach enhanced the FOSs’ purity by reducing the monosaccharide content while increasing the FOSs concentration. The immobilized FTase converts the remaining sucrose into FOSs.

Membrane bioreactors include conventional bioreactors and membrane-filtering devices that retain the biocatalyst [44]. EMRs have also been used in the bioconversion of large-molecular weight compounds using a membrane-integrated enzyme. In these devices, membranes are used to compartmentalize or immobilize biocatalysts. These EMRs can eliminate enzyme-free products in situ and maximize enzyme reusability through the activity of free and soluble enzymes. In one study, a filtering model was utilized to identify components that functioned as biocatalysts for continuous enzymatic FOS production. The study also investigated the relationship among separation efficiency, flow, and the membrane cutoff rating [44].

**Figure 2:**
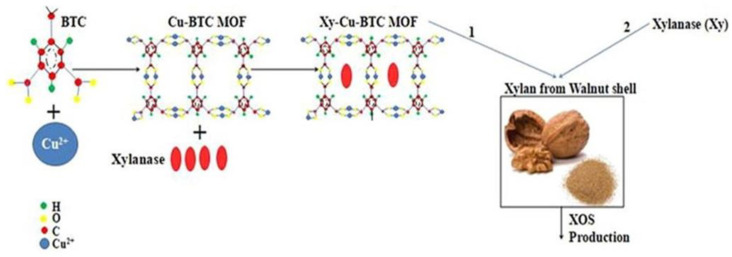
Production of xylotetrose and xylopentose from waste walnut shells using free xylanase (Xy) and MOF immobilized xylanase (Xy-Cu-BTC) [52].

Xylooligosaccharides (XOSs) are oligosaccharides composed of xylose sugars that can be isolated from plant hemicelluloses, such as those present in shoots and seeds. The action of the enzyme xylanase on hemicellulose produces xylose and XOS as by-products [50,51]. Kaushal et al. reported that xylan, the extraction of leftover walnut shells, can increase the degree of XOS polymerization. Free xylanase was immobilized in a copper-based metal–organic framework using green synthesis. Both the free and immobilized forms of xylanase may promote the biotransformation of xylan into XOSs (Figure 2). The total yield of xylan has been reported to approximately 87.4%, and it is accompanied by the production of high concentrations of xylotetrose (X4) and xylopentose (X5). XOS has been reported to be composed of 4.1% X4 and 60.57% X5 in a free enzyme system, and the yield of X4 and X5 was increased to 11.8% and 64.2%, respectively, in an enzyme immobilized system [52].

The enzyme xylose is used to obtain XOS from beechwood xylan. For monitoring, an inline viscometer was used to assess hydrolysis, and the process was performed in EMRs in batch, continuous, and semi-continuous modes. In all modes, beechwood xylan was added under high temperature (50 °C) and an acidic pH (5.8). Increasing the hydrolysis rate for xylan production thus requires the use of a suitable enzyme. Additionally, three types of gut bacteria were used in in vitro fermentation to confirm the prebiotic properties of the obtained XOS, which had an average degree of polymerization [53]. Gentiooligosaccharides, which can be produced by the action of β-glucosidases, are used as prebiotics in alternative food sectors [39]. Gentiooligosaccharides were first synthesized using Blg163, which required the lowest amount of β-glucosidase. The commercial production of gentiooligosaccharides using β-glucosidase is challenging owing to high energy requirements and increased enzyme demand. The reverse hydrolysis of glucose using Blg163 at pH 7.0 and 40 °C yielded 43.02 g·L^−1^ of gentiooligosaccharides with a conversion rate of 5.38 ± 0.40%. Blg163 was reported to transglycosylate the mixed substrate containing glucose and cellobiose, and it produced 70.34 g·L^−1^ of gentiooligosaccharides at a conversion rate of 15.63% [39].

Chitooligosaccharides are chitin derivatives and by-products of chitosan enzymatic hydrolysis [54]. In this study, the native enzyme, chitosanase Csn75, was combined with a curdlan-specific carbohydrate-binding module (CBM), and *Pichia pastoris* was used to successfully produce recombinase Csn75-CBM. This system facilitated the purification and immobilization of chitosanase on a curdlan packed bed reactor. The highest enzyme adsorption capacity (39.59 mg/g curdlan) was achieved in the packed-bed reactor. When the enzyme-to-chitosan ratio and chitosan flow velocity were adjusted, higher hydrolysis was observed at 2–5 degrees of polymerization. The use of EMRs for the production of valuable products from different raw materials is summarized in Table 1.

### 3.2. Oligodextran Production 

**Figure 3:**
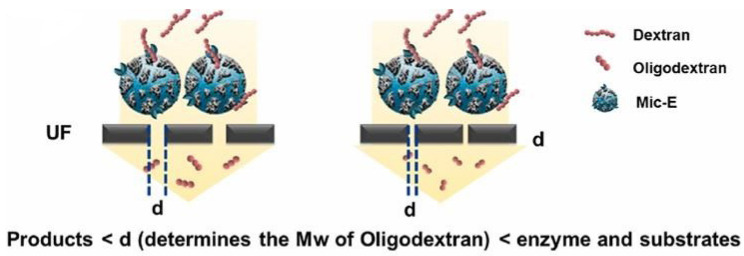
Schematic representation of ultrafiltration (UF)-based enzyme membrane bioreactor (EMR) production process [57].

High-performance enzyme reactors are necessary for stable and effective bioconversion processes. However, this remains challenging, as only a few EMR designs simultaneously possess a high enzyme load capacity, appropriate mixing, and adequate mass flow [57,59]. The advantages of packed bed reactors and EMRs can be combined to overcome these limitations. The EMR model for enzyme-loaded microsphere UF is employed (Figure 3). Invertase and dextranase are two enzymes used to hydrolyze sucrose and dextran, respectively, to obtain glucose and oligodextran. Furthermore, microspheres can reduce membrane fouling caused by unbound enzymes and increase the contact area between the enzymes and substrates. Consequently, EMRs with free and immobilized enzymes (free and Mic-UF) converted sucrose at a higher rate than EMRs with free invertase, with little loss in sucrose conversion being observed after 24 h of continuous operation. With the selection of an Mic-UF EMR membrane with an appropriate MWCO, the dextranase-based Mic-UF EMR achieves oligodextrans with appropriate molecular weights and narrow molecular weight distributions.

**Figure 4:**
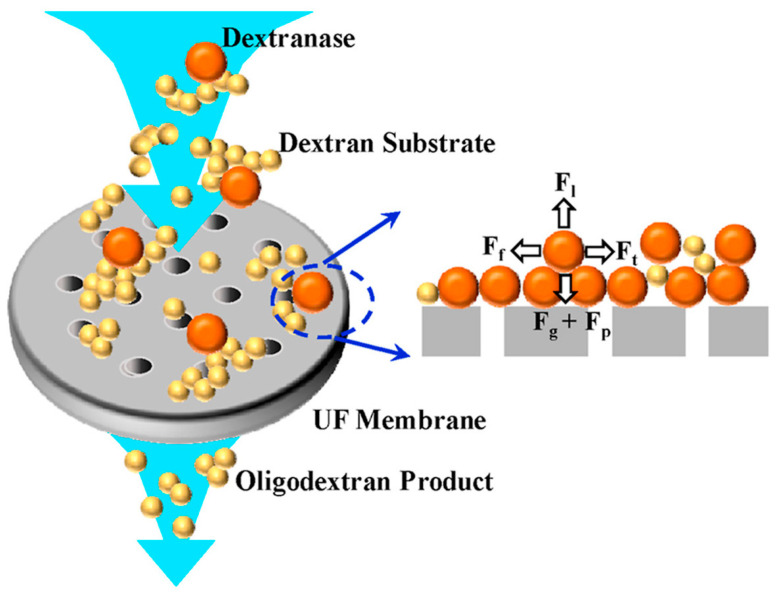
Illustration of an enzyme membrane bioreactor (EMR) for oligodextran production [36].

A mathematical model has been proposed to simulate the filtration performance of an EMR system for oligodextran production [36]. The model was validated via bench-scale experiments, and the effects of membrane properties and operating conditions on EMR performance were evaluated. A polyethersulfone UF membrane with an MWCO of 20 kDa (PES20) was designed to retain or separate molecules and particles with molecular weights greater than 20 kDa. The uniform membrane pore size distribution and higher membrane porosity significantly enhanced the filtration efficiency and product quality. However, a higher permeate flux may result in decreased permeate concentration owing to factors such as denser fouling layers, shorter hydrolysis retention time, and increased water convection transport (dilution effect). In the water-feeding mode, optimal performance can be achieved by applying a higher driving force and lower agitation speed to reduce fouling tendencies. However, more severe membrane fouling occurs in the substrate feeding mode, thus necessitating intense agitation and moderate operating pressure to enhance the production efficiency. This study provides valuable insights into the technical feasibility and practical constraints of EMR systems and offers guidance for their further development (Figure 4).

**Figure 5:**
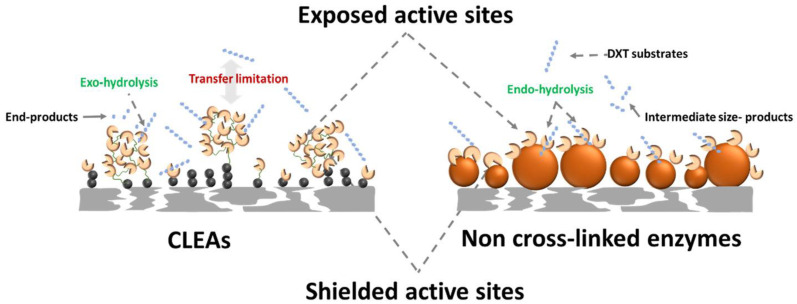
Accessibility of large dextran molecules to the enzyme active sites on the membrane surface in enzyme membrane reactors (EMRs), resulting in an exo- or endohydrolysis pattern [56].

When the PDA-modified membrane of an EMR is crosslinked with dextranase, dextran undergoes conversion through an exohydrolysis pattern. This is due to the more efficient immobilization of enzymes on the membrane surfaces in the fouling-induced mode. However, dextranases that are not crosslinked show a normal endohydrolysis pattern (Figure 5). In both systems, the dextran yield is increased [56]. When the dextranase is aggregated or crosslinked on the membrane surface, the active sites of the enzyme may become partially or fully shielded by the aggregation or crosslinking structure. The aggregation of dextranase molecules can lead to the formation of larger enzyme clusters or aggregates, which may limit the access of large dextran molecules to active sites. Consequently, the hydrolytic activity of the aggregated dextranase may be reduced compared to that of the non-crosslinked form. The shielding effect of aggregated dextranase can affect the type of hydrolysis that occurs. The shielding of active sites in aggregated dextranase may favor exohydrolysis, as limited access to the internal sites of dextran molecules may hinder endohydrolysis [56].

### 3.3. Oligorutin Production

**Figure 6:**
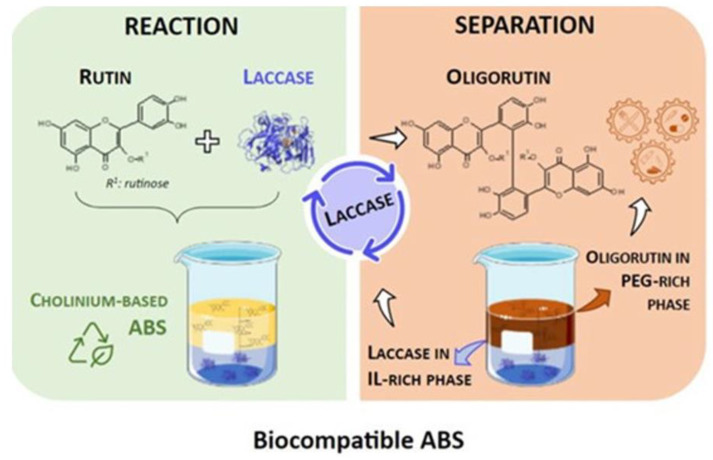
Integrated biocatalytic platform based on aqueous biphasic systems for the sustainable oligomerization of rutin [60].

Oligorutin is an oligomeric flavonoid produced via the action of peroxidases and laccases. Oligorutin exhibits bioactive and antioxidant properties. In a previous study, a UF EMR was designed to synthesize oligorutin, which can be reused in further cycles [30], and remarkable laccase stability was observed after 24 h cycles, with no extra enzyme addition. Oligorutin demonstrated up to 1620 times water solubility, 24 times iron-chelating activity, and approximately 80% reduced ferric-reducing antioxidant power. Similarly, EMRs with an integrated reaction separation system facilitated the oligomerization of rutin by laccase. This unique biphasic system is composed of polyethylene glycol-600 and cholinium dihydrogen phosphate, which are ionic liquids (Figure 6) [60].

### 3.4. Protein Hydrolysates Production

Because protein hydrolysates are a functional source of biologically active peptides, their production in a conventional enzymatic batch reactor process requires large amounts of enzymes and has high energy demands and labor costs, which are drawbacks of enzymatic batch methods [16,61]. Sodium caseinate hydrolysate with better antioxidant activity and decreased bitterness in a continuous system has been reported to result in a functionally improved permeate with enhanced antioxidative properties, and a two-step EMR technique has been developed using endopeptidase Sternzym BP 25201. Separating the endo and exopeptidases Into two EMR phases reduces the impact of the peptidases on each another, and it ensures consistent qualities in the resulting product, such as the degree of hydrolysis, antioxidative properties, and flavor. The process settings used were ideal for stable product development over three days. This method is superior to batch processes in terms of productivity, enzyme usage, and runtime. Furthermore, the bioactive potential of the protein hydrolysate from black soldier flies (BSF) larvae using bromelain in an enzymatic hydrolysis method has been investigated [62], and it was reported that a protein hydrolysate yield of 10.70% (by a weight basis) and productivity of 21 mg/L/batch were achieved.

### 3.5. Biohydrogen Production

**Figure 7:**
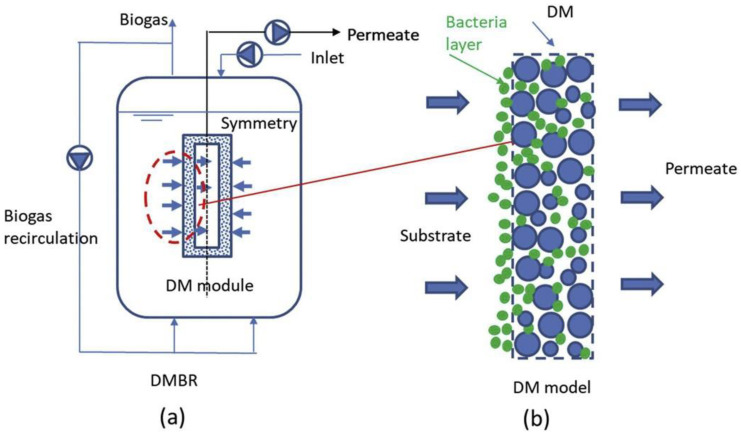
(**a**) The schematic diagram for the formation and fouling of dynamic membrane (DM): configurations of submerged dynamic membrane bioreactor (DMBR), and (**b**) dynamic membrane (DM) model [14].

Sustainable biohydrogen has excellent potential for restoring fossil fuels. Efficient dark fermentative biohydrogen generation in active membrane reactors depends on membrane microbial activity management strategies [14,63,64,65]. A complex EMR model with biofilm growth, dynamic membrane creation, and dark fermentative hydrogen production was created using lattice Boltzmann and cellular automata platforms. Dynamic membrane creation involves three phases: namely, the growth of stable biofilm, maximum stable biofilm biomass, and initially deposited bacteria. These findings demonstrate that biohydrogen extraction and biofilm formation could be considerably affected by porous twisted channels in the dynamic membrane (Figure 7) [14]. Dynamic membranes are thin layers formed by microbial growth on the surfaces of porous support materials that act as filters and separators in bioreactors. The authors developed a mathematical model to simulate and predict the growth of microbial communities in dynamic membrane systems. The model was designed to understand microbial behavior and assess its impact on biohydrogen production performance. By integrating biological and physical factors, the model considers various parameters, such as substrate concentration, microbial growth rate, biomass density, and other relevant variables. The model simulation allowed researchers to analyze the dynamic behavior of the microbial community and predict its impact on the performance of the biohydrogen production bioreactor [14]. The porosity of a structure can increases during biohydrogen generation, and a permeable system and high inflow velocity are critical operating parameters for continuous biohydrogen production. A single chamber microbial electrolysis cell (MEC) method for biohydrogen production has been previously described. Water hyacinth can potentially serve as the only substrate required for dark fermentation using the MEC technique [66].

### 3.6. N-Acetylneuraminic Acid Production

*N*-acyl-d-glucosamine 2-epimerase and *N*-acetylneuraminate lyase have been used as models for the two-step synthesis of *N*-acetylneuraminic acid, which is a high value pharmaceutical starting material [40]. The model analysis facilitated an in silico process optimization of the reaction conditions and mode of operation. Thus, a combined strategy that applies parallel EMRs and mechanistic models on a milliliter scale was suggested to be applicable for the first time to help accelerate enzymatic production processes. Furthermore, the mass production of *N*-acetylneuraminic acid could potentially be accomplished using recombinant *Escherichia coli* simultaneously expressing *N*-acetyl-d-glucosamine-2-epimerase and *N*-acetyl-d-neuraminic acid aldolase as biocatalysts [67].

### 3.7. Sugar Production

Various types of biomasses can be used to produce sugar. Pectic oligosaccharides are the simplest forms of sugar, and pectin, which is obtained from sugar beet pulp, is a desirable source of pectic oligosaccharides [55]. In continuous cross-flow EMRs, pectic oligosaccharides are produced using inexpensive crude enzymes, and batch and semi-continuous processes are used to determine the appropriate enzyme concentrations and evaluate the appropriateness of filtering. 

An alternative method for sugar production involves depolymerizing lignocellulosic biomass [15]. Optimal sugar production depends on several factors, including enzyme loading, pH, temperature, and reaction time. In one study, pineapple leaf hydrolysate was used as a substrate for sugar production. β-xylosidase was used to hydrolyze the feed substrate under different working conditions using the one-factor-at-a-time technique. The optimal operating conditions of enzymatic hydrolysis were applied to the integrated enzyme membrane system, wherein the simultaneous reaction and filtering resulted in a much higher sugar yield (293.94%) [15].

## 4. Bioconversion or Transformation Using EMR

Larger molecules (proteins, polysaccharides, and lipids) can be bioconverted or biotransformed through hydrolysis into functional products and bioactive peptides that find application in the food, pharmaceutical, and biomedical industries [17,68]. The differences between EMRs used in production and bioconversion lie in the specific requirements of the desired product, compatibility of the membrane material with enzymes, and optimization of operating conditions to achieve the desired outcome. The membrane material, structure, module, and operating parameters are selected based on the specific application and objectives of the EMR. Bioconversion is generally conducted in the presence of optimum substrate/product concentrations, where the biocatalyst exhibits optimal activity and stability, thus maximizing productivity [69]. In this regard, EMRs can be considered effective macromolecular hydrolysis systems [70]. As the membrane continuously maintains the substrate and enzyme within the reactor, substrate conversion is improved, and product purification becomes more feasible. Thus, the discussion in this review also focuses on recent information related to the bioconversion of chitin, lignin, gluten, and lactose in EMR using various membranes as well as their advantages and disadvantages.

### 4.1. Conversion of Chitin

**Figure 8:**
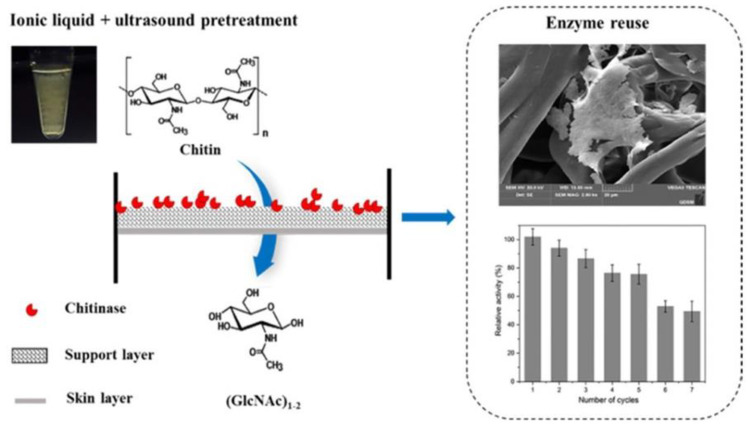
An immobilized enzyme membrane reactor for the efficient and sustainable conversion of ionic liquid/ultrasound pretreated chitin [42].

Chitin, produced from crustacean waste, is the second-most common renewable biopolymer found in nature [42], and it is used to produce functional oligosaccharides that find application in the chemical, pharmaceutical, culinary, and agricultural industries. Therefore, research has been conducted on the hydrolysis of chitin into value-added chemicals. An EMR for immobilized enzymes was developed and used to hydrolyze chitin for the first time, wherein chitinase from *Streptomyces albolongus* ATCC 27414 (SaChiA4) was immobilized by adsorption on the regenerated cellulose UF support layer membrane (Figure 8). The complete conversion of chitin was observed when it was pretreated with 1-ethyl-3-methylimidazolium acetate, which was followed by ultrasonication. The maximum conversion rate of chitin was reported to be 75.60% even after five cycles at 40 °C. This shows that chitin can be converted in an eco-friendly and effective manner using a biocatalytic UF membrane reactor [42]. 

### 4.2. Conversion of Gluten

Gluten is a cereal protein, and approximately 85% of wheat gluten, the most common type, is composed of protein. The hydrolysates of this grain protein are widely used in culinary products. Various methods are used in protein bioconversion. For the biotransformation of gluten, EMR techniques can be used to immobilize enzymes on the membrane and convert the product using inexpensive methods. In a previous study, wheat gluten was continuously hydrolyzed using the proteolytic enzyme flavourzyme^®^ in an EMR [71]. A steady yield of 6.33 g h^−1^ L^−1^ was obtained after 96 h of long-term hydrolysis at 37 °C at pH 7.5 in the presence of 8% (*v*/*v*) ethanol at a substrate concentration of 100 g L^−1^. Using the EMR approach, enzyme productivity was increased by 459% compared to a batch procedure, and 30 min of discontinuous clearing of accumulated dry matter was required every 24 h for enzymes to be reused.

### 4.3. Bioconversion of Lactose 

Lactose is converted into epilactose, a prebiotic, by the enzyme 2-epimerase (CE). Several enzymes have been designated as members of the recently found cellobiose 2-epimerase enzyme family. In one study, recombinant 2-epimerase was generated by inserting the products of the CE genes from the mesophilic bacteria *Cellulosilyticum lentocellum* and *Dysgonomonas gadei* into *E. coli* and purifying the obtained enzymes [72]. Recombinant enzymes are used in EMRs for the epimerization of cellobiose and lactose into glucosylmannose and epilactose, respectively. It was reported that the CE from *C. lentocellum* and that from *D. gadei* both showed the highest lactose epimerization activity at pH 8–7.5 at 40 °C temperature, respectively. A batch approach or a continuously run EMR procedure was used to achieve the bioconversion of lactose in milk ultrafiltrate. The epilactose yield in the batch process was 29.9% within 28 h, but that in the continuous EMR process was lower. However, enzyme production was six times higher in the continuous process. It has been reported that milk ultrafiltrate may be improved into a high-value dairy product through the enzymatic conversion of lactose using the EMR process.

### 4.4. Biological Aging of Beer

**Figure 9:**
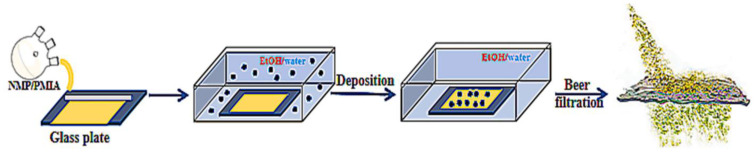
Schematic diagram of poly (m-phthaloyl-m-phenylenediamine) (PMIA) biocatalysis membrane modification by pulluanase@chitosan porous beads (PULL@CPB) [73].

In the beverage industry, the ripening of beer without compromising beer quality is of utmost importance. If the alcohol-to-ester ratio of beer is between 3.5 and 4.5:1, it is considered high-quality beer. In a previous study, a novel membrane known as Pullulanase@Chitosan Porous Beads/Poly (m-phthaloyl-m-phenylenediamine) (PULL@CPB/PMIA) was developed using a self-adhesive method to immobilize the enzyme (PULL@CPB) onto the PMIA membrane [73]. This combination exhibited favorable separation and biocatalytic properties, enabling the efficient refinement of protoplasmic beer and reducing the alcohol-to-ester ratio. This study found that increasing the amount of PULL@CPB and the alcohol/water ratio in the coagulation bath enhanced the pullulanase load on the membrane surface, although excessive addition resulted in reduced enzyme activity. Optimal conditions were achieved with 0.5 g·L^−1^ of PULL@CPB and an EtOH/water ratio of 60%, yielding the highest relative activity of pullulanase at 91.7% of the initial activity. The PULL@CPB/PMIA membrane demonstrated excellent interception rates of protein and macromolecular β-glucan, reaching 92.2% and 87.3%, respectively, under a beer flux condition of 162.3 L·m^−2^·h^−1^. The immobilized pullulanase retained 70.8% of its activity after ten continuous uses, highlighting the durability and stability of the PMIA membrane as an effective carrier for pullulanase. The PMIA membrane has a superior ability to transport pullulanase, and it has various applications in food, medicine, and other industries (Figure 9) [73].

### 4.5. Conversion of Lignin 

Lignocellulose is a renewable biomass that is typically used to produce fine chemicals and biofuels. Lignin is mainly used to produce value-added chemicals; however, its depolymerization is energy consuming and ecologically unfavorable. In this regard, the enzymatic conversion of lignin has attracted the attention of researchers, and the emerging application of the EMR solves the valorization and hydrolysis of lignin. The benefits of this system include enhanced reaction cycles, reduced product inhibition, improved stability, and adaptability to complex enzymatic processes. Lignin can also be synthesized in the EMR of a continuously stirred-tank reactor using an external ceramic cross-flow UF membrane immobilized with a lignin-degrading enzyme (heme peroxidase). Filtration membrane fouling and H_2_O_2_-induced irreversible enzyme inhibition are the two major limitations of this system, which can be addressed using a continuous operation method. 

The use of guaiacol glycerol ether (GGE) in EMRs holds great promise for various applications. In a recent study, a GGE solution (3.1 mM in 100 mL of deionized UF (DIUF) water) was efficiently processed using a polyvinylidene fluoride (PVDF) membrane modified with polyacrylic acid (PAA) [74]. A layer-by-layer assembly technique was used to enhance the membrane functionality. Initially, the PAA-modified membrane retained its negative charge even after treatment with DIUF at pH 6. This enabled the introduction of a positively charged electrolyte by permeating a solution of poly (allylamine hydrochloride) (PAH) with a concentration of 45 µM at pH 3.9. The electrostatic interactions between the oppositely charged PAA and PAH resulted in the immobilization of PAH within the membrane pores. Sequential immobilization of the enzymes was achieved by permeating solutions of laccase, horseradish peroxidase, and glucose oxidase individually, each at a concentration of 100 ppm (10 mg/100 mL), in DIUF water at pH 6. This process led to the formation of multi-enzyme-incorporated membrane reactors. These reactors showed good performance, as they remained active for over 30 days during storage at 4 °C. Moreover, the reactors exhibited excellent stability and efficiency during repeated operation cycles, with each cycle lasting for 5–6 h. Notably, the multienzyme immobilized membranes demonstrated high efficacy, degrading over 90% of the initial feed within a residence time of approximately 22 s. Analysis of the GGE conversion products revealed the formation of oligomeric oxidation products upon reaction with peroxidase, which could pose a potential risk to membrane bioreactors. However, the presence of laccase enzymes in the multienzymatic membranes facilitates the further degradation of these oxidation products, thus mitigating their potential adverse effects.

Furthermore, EMRs are used in biorefineries of lignocellulose biomass [75]. Cellulolytic enzymes from *Trichoderma harzanium* BPGF1 were directly encapsulated in a PVDF membrane during the hydrolysis of lignocellulose biomass. The enzyme-encapsulated membranes used acid-treated corncobs as feed and showed a higher reaction speed and glucose yields as well as enhanced hydrolytic efficiency.

### 4.6. Conversion of Hesperidin

**Figure 10:**
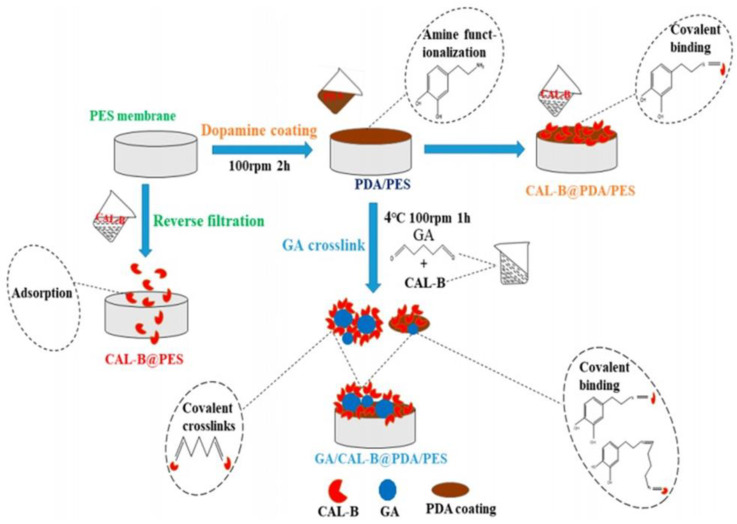
Schematic diagram for bioinspired lipase immobilized membrane (BLIM) fabrication [76].

Flavonoids are plant secondary metabolites that are phenolic compounds with strong antioxidant capacity [76]. Orange, lemon, and many other citrus fruits are rich in flavonoids and antioxidants, and they exhibit anticancer properties; however, the body cannot absorb flavonoids because of their poor liposolubility. One study focused on the development of a bioinspired lipase-immobilized membrane to enhance hesperidin lipophilization. Hesperidin is a natural compound found in citrus fruits that has various health benefits. By incorporating immobilized lipase into the membrane, this study explored the enhancement of hesperidin lipophilization, which involves the attachment of a lipid moiety to the hesperidin molecule. Bioinspired lipase-immobilized membranes (BLIMs) were prepared using a PDA coating followed by GA co-deposition, as shown in Figure 10. The BLIMs existed in three distinct forms: CAL-B@PES, CAL-B@PDA/PES, and GA/CAL-B@PDA/PES. Reverse filtering, PDA coating, and GA crosslinking were used to construct the BLIMs by fixing *Candida antarctica* lipase B (CAL-B) on the membrane. Compared to the other two BLIMs, GA/CAL-B@PDA/PES exhibited the highest enzyme activity, the most effective rate of hesperidin esterification, and the greatest resistance to environmental fluctuations. The optimal working parameters for the production of GA/CAL-B@PDA/PES were CAL-B concentration at the operating pressure, GA concentration, and crosslinking.

### 4.7. Conversion of Inulin 

The agricultural by-product inulin is a natural polymer. DFA III, a cyclic disaccharide with two fructose units, is a high-value compound produced during the bioconversion of inulin. Inulin is primarily produced using the enzyme inulin fructo-transferase [45]. In the food and pharmaceutical industries, DFA III is considered a promising food additive because it acts as a low-calorie sweetener and promotes calcium absorption in the small and large intestines. Conventional enzyme reactor methods produce DFA III, but they have several drawbacks owing to lengthy, labor-intensive procedures that require many enzymes. It has been reported that inulin can be converted using an EMR linked to a nanofiltration membrane system that can extract large concentrations of DFA III [45]. Through this process, IFTases can be recycled and reused in EMR. This system shows that the nanofiltration membrane attached to the EMR with MWCOs of 5 kDa and 150 Da increased DFA III production to approximately 400 g/L. This may also provide theoretical information for scaling up industrial output. The researchers achieved stable and continuous operation for eight runs, indicating the reliability and persistence of the system over time. Similarly, the bacterium *Arthrobacter chlorophenolicus* A6 cloned and overexpressed in *E. coli* can be used to manufacture inulin [41]. Recombinant IFTase was isolated using gel filtration and SDS PAGE gel electrophoresis. In this process, the enzyme was shown to be homotrimeric with three identical subunits. The catalytic activity of the enzyme peaked at 65 °C and pH 5.5. Minor products such as sucrose, 1-kestose, and nystose were also produced after complete inulin hydrolysis for DFA III production. The Y-zeolite adsorption process was used to convert inulin and purify DFA III. The molecular modeling of DFA enables its purification without changing its conformation. After crystallization, the selective adsorption of DFA III from the residue and desorption with ethanol were possible [77]. The use of enzyme membrane bioreactors for the bioconversion and transformation of different raw materials into valuable products is summarized in Table 2.

## 5. Conclusions and Future Prospects

EMRs can be used in the production of valuable industrial products, such as oligosaccharides, oligodextrans, inulin, and sugars, which are widely used in biopharmaceutical, food, and agroindustries, and they are cost-effective but time consuming. Moreover, EMRs are used for the optimal recovery of the starch component. FTases are widely used in EMRs because they generate FOS from sucrose. FTases bind to acceptors, such as sucrose or 1-kestose, elongate the chain by adding a fructosyl functional group derived from sucrose to the reducing end, and release extra glucose as a by-product. Further research is required to produce FOS on an industrial scale to improve the FOS yield and remove by-products and unreacted substrates. Dextranase is an enzyme widely used for the production of oligodextran via the conversion of polydextran to oligodextran. A membrane is used as a selective sieve to obtain oligodextran products with intermediate molecular weights. Membrane bioreactor technology is widely used in various industrial wastewater treatments owing to its advantages over conventional technologies. Therefore, extensive research is required to determine the optimal operating modes and reaction conditions. In addition to using mechanistic process models, laboratory equipment minimization and parallelization can accelerate bioprocess optimization. Continuously running EMRs may provide a comparable and reproducible technique in the future, enabling the long-term functioning of enzymes. Additional research should be conducted to assess the effectiveness of multienzyme membrane bioreactors. The in silico process optimization of the reaction conditions and mode of operation must be investigated to enable the extraction of valuable products from agricultural waste. The future prospects for EMRs are promising as they offer several advantages in various industries. EMRs can potentially be used in sustainable and environmentally friendly biocatalytic processes, and they can further enhance the efficiency and stability of biocatalytic reactions, making them attractive candidates for green chemical applications. They can also be used to produce pharmaceutical intermediates, natural products, and chiral compounds. EMRs have potential biomedical applications, including as enzymatic sensors, drug delivery systems, and biofuel cells. The precise control of enzymatic reactions offered by EMRs can facilitate the development of bio-sensors for the detection of specific molecules or biomarkers. Additionally, EMRs can be used for the controlled release of drugs or therapeutic agents to improve their efficacy and minimize their side effects as well as for wastewater treatment and environmental remediation. This can be achieved by immobilizing enzymes on membranes to degrade pollutants or convert harmful compounds into less toxic forms. EMRs can efficiently remove contaminants, improve enzyme stability, and reduce fouling, thus contributing to the development of sustainable water treatment solutions. To realize these future prospects, current research should focus on developing advanced membrane materials, enzyme immobilization techniques, reactor designs, and optimizing operating conditions. Integration with emerging technologies such as microfluidics, nanotechnology, and genetic engineering may further enhance the performance and versatility of EMRs for various applications.

## Figures and Tables

**Table 1:** Enzyme membrane bioreactors and its production rate for valuable product.

Reactants	Name of the Enzyme	Sources	Product	Yield		Reference
				In %	g·L^−1^	
GOS syntheses, lactose solutions, cellulose acetate membranes	β Galactosidase	Lactose	Galactooligosaccharide	60%		[5]
PVDF hollow fiber with an epoxy resin-sealed in the flux inlet of the membrane’s bottom	Spezyme Fred enzymes from *Bacillus licheniformis* for liquefaction and Optidex L-400 from *Aspergillus niger* for saccharification	Pure wheat starch with a starch content of 95.1%	Glucose Syrups		73.6 ± 4.3 g glucose L^−1^	[47]
Enzyme membrane reactor, FOS, fructosyltransferase (1-FFT)	Aspergillus terreus fructosyltransferase (1-FFT)	Cell-free suspension of *Kluyveromyces lactis* GG799 strain for enzyme production	Fructooligosaccharide	-----		[48]
Soluble Biolacta N5, a *Bacillus circulans*-derived commercial enzyme	Biolacta N5 (derived from *Bacillus circulans*)	Lactose	Galactooligosaccharide	38%		[46]
L-cysteine grafted polydopamine-coated membrane	FTases	Sucrose	Fructooligosaccharide	51.4 to 92.3%		[49]
Sugar beet pulp + nitric acid	Viscozyme	Sugar beet pulp	Pectic oligosaccharides	82.9 ± 9.9%		[55]
Ceramic membrane EMR	FTases	Sucrose (600 g·L^−1^)	Fructooligosaccharide	10%		[44]
Transfructosylation of Sucrose by *Pichia pastoris*	FTases	Buffered glycerol complex medium (BMGY)	Fructooligosaccharides		270 g·L^−1^	[37]
Cu-BTC MOF by using CuCl_2_ along with BTC (benzene-1, 3, 5-tricarboxylate)	Xylanase (Xy)	Waste walnut shells	Xylotetrose and xylopentose	11.8% X4 and 64.2% X5		[52]
Methacrylate polymer Lifetech ECR8285 has butyl and epoxy groups functionalized on its surface	FTases	Sucrose	Fructooligosaccharide	92.1%	130–170 g·L^−1^	[43]
Beechwood xylan (BX)	CTec2 (a mixture of cellulase, glucosidase, xylanase, xylosidase and arabinosidase activity)	Beechwood xylan	Xylo-oligosaccharides (XOs) and xylose	48%		[53]
Reverse hydrolysis of glucose (Blg163)	β-glucosidase gene from coral microbial metagenome produced in *E. coli*	Cellobiose and glucose	Gentiooligosaccharides		70.34 ± 2.20 g·L^−1^	[39]
Carbohydrate-binding module + Csn75	Chitosanase Csn75	-----	Chitooligosaccharides	97.75%		[54]
Enzymatic hydrolysis of enzymatic reactor ultrafiltration	Dextranase	Dextran	Oligodextran	-----		[36]
Polydopamine → tannic acid + hydrolyzable 3-amino-propyltriethoxysilane, crosslinked and non-crosslinked dextranase	Dextranase	Dextran	Oligodextran	-----		[56]
Enzymatic packed bed membrane reactor, PBR, EMR	Invertase and dextranase	Sucrose	Glucose and oligodextran	Sucrose conversion rate 85%		[57]
Pineapple leaves + b-xylosidase	β-xylosidase	Pineapple residues (leaves)	Sugar	Reaction and filtration rate much higher than (293.94%) reaction alone (32.23%)		[15]
Polysaccharide, protein	Catalytic degradation	-----	Oligomers and polymers	55.8% to 92.3%		[58]
Oligomerization reactions in organic-free reaction media	Peroxidases and laccases	Rutin	Oligorutin	-----		[30]
Two-step EMBR process	Peptidases	Sodium caseinate	Antioxidative casein hydrolysates	Antioxidativity: +39%		[16]
Three phases of dynamic membrane formation	-----	-----	Dark fermentative hydrogen production	16.4%		[14]
Parallel enzyme membrane reactors, cascades of EMRs	*N*-acyl-d-glucosamine 2-epimerase and an *N*-acetylneuraminate lyase.	-----	*N*-acetylneuraminic acid (Neu5Ac)	-----		[40]

Abbreviation: GOS, galactooligosaccharide; STR, stirred tank reactor; ISPR, in situ product recovery; FTases, fructosyltransferases; FOS, fructooligosaccharide; PBR, packed bed reactor; SBP, sugar beet pulp; XOS, xylooligosaccharide; EMRS, enzymatic membrane bioreactor system; BX, beechwood xylan; PVDF, polyvinylidene fluoride membrane; Csn75, chitosanase 75; EMR, enzyme membrane bioreactor; DFAs, fructose dianhydride; In%, percentage of yield.

**Table 2:** Bioconversion process using enzyme membrane bioreactors and its conversion rate.

Reactants	Name of the Enzyme	Sources	Product	Yield		Reference
				In %	g·L^−1^	
Ceramic membrane filtration	Yeast (*Kluyveromyces lactis*)	-----	*K. lactis* biomass	-----	-----	[78]
Encapsulation of cellulolytic enzymes isolated from *Trichoderma harzanium* BPGF1 in polyvinylidene fluoride membrane	*Trichoderma harzanium* BPGF1	Lignocellulosic biomass	Lignocellulosic biomass	72.46 ± 2.4%		[75]
Hydrophobic Y-zeolites (adsorb DFAs from aqueous solution), depolymerization of inulin	Exo-inulinase	Freeze-died molasses	Difructose dianhydrides III (α-D-fructofuranose β-D-fructofuranose 1,2′:2,3′-dianhydride; αf(1,2′:2,3′)βf)	-----		[77]
Enzyme membrane reactor nanofiltration	Inulin fructotransferase	Inulin	Difructosan anhydride III		400 g·L^−1^	[45]
Cloned Arthrobacter chlorophenolicus A6 is overexpressed on *E. coli*, chromatography, sodium dodecyl sulfate-polyacrylamide gel electrophoresis	Inulin fructotransferase	Inulin	Difructose anhydride III	Sucrose (81%), 1-kestose (72%), nystose (67%)		[41]
EMRS + external ceramic crossflow ultrafiltration membrane	Ligninolytic heme peroxidases	Ligninsulfonates (LSs) and kraft lignins	Lignin	-----		[79]
Fabricated through layer-by-layer assembly, pH-responsive membranes, polymer membrane reactor	Glucose oxidase, peroxidase and laccase	Guaiacylglycerol-β-guaiacyl ether	Lignin, fabrication of multienzyme.	Loss 12% of initial activity		[74]
Cellulose ultrafiltration membrane, chitinase from *Streptomycesalbolongus*	Chitinase	Chitin	Convert chitin	Chitin conversion rate of 75.60%		[42]
Reverse filtration, crosslinking of GA and PDA coating	*Candida antarctica* lipase B (CAL-B)	-----	Hesperidin esterification		73.6 ± 4.3 g glucose L^−1^	[76]
Food-grade EMR process, 8% ethanol	Ethanol	Flavourzyme wheat gluten	Converted wheat gluten		6.33 g h^−1^ L^−1^	[71]
Lactose + cellobiose 2-epimerases	Cellobiose 2-epimerases	Milk ultrafiltrate containing lactose	Epilactose	Epilactose production was lower (18.5%)		[72]
PMIA membrane	Pulluanase @chitosan	-----	Biological aging of beer	70.8%		[73]

Abbreviation: PBR, packed bed reactor; SBP, sugar beet pulp; XOS, xylooligosaccharide; EMRS, enzymatic membrane bioreactor system; BX, beechwood xylan; PVDF, polyvinylidene fluoride membrane; Csn75, chitosanase 75; EMR, enzymatic membrane bioreactor, In%, percentage of yield.

## Data Availability

Not applicable.
